# Characterization of Individual Projections Reveal That Neuromasts of the Zebrafish Lateral Line are Innervated by Multiple Inhibitory Efferent Cells

**DOI:** 10.3389/fnana.2021.666109

**Published:** 2021-06-21

**Authors:** Remy Manuel, Ana Belen Iglesias Gonzalez, Judith Habicher, Harmen Kornelis Koning, Henrik Boije

**Affiliations:** Department Neuroscience, Uppsala University, Uppsala, Sweden

**Keywords:** *Danio rerio*, sensory modulation, neuromast, *dmrt3a*, CEN, REN, ROLE, RELL

## Abstract

The zebrafish lateral line is a sensory system used to detect changes in water flow. It is comprized of clusters of superficial hair cells called neuromasts. Modulation occurs via excitatory and inhibitory efferent neurons located in the brain. Using mosaic transgenic labeling we provide an anatomical overview of the lateral line projections made by individual inhibitory efferent neurons in 5-day old zebrafish larvae. For each hemisphere we estimate there to be six inhibitory efferent neurons located in two different nuclei. Three distinct cell types were classified based on their projections; to the anterior lateral line around the head, to the posterior lateral line along the body, or to both. Our analyses corroborate previous studies employing back-fills, but our transgenic labeling allowed a more thorough characterization of their morphology. We found that individual inhibitory efferent cells connect to multiple neuromasts and that a single neuromast is connected by multiple inhibitory efferent cells. The efferent axons project to the sensory ganglia and follow the sensory axon tract along the lateral line. Time-lapse imaging revealed that inhibitory efferent axons do not migrate with the primordium as the primary sensory afferent does, but follow with an 8–14 h lag. These data bring new insights into the formation of a sensory circuit and support the hypothesis that different classes of inhibitory efferent cells have different functions. Our findings provide a foundation for future studies focussed toward unraveling how and when sensory perception is modulated by different efferent cells.

## Introduction

Sensory systems provide information regarding the environment that is translated into behaviors aimed at increasing an organism’s chance of survival. To allow for adaptation and to distinguish between self-inflicted or external stimulation, there must be filtering or modulation of the sensory input. Modulation can be found in many biological systems, such as hearing (Whitfield, 2002), vision (Sheynin et al., 2020), and pain (Damien et al., 2018). The modulation can provide inhibitory feedback signals that reduce the response to constant excitation, thereby protecting the system from overload (i.e., habituation; Groves and Thompson, 1970; Ramaswami, 2014), or supply feedforward inhibition, desensitizing sensory systems to self-induced activation (Lunsford et al., 2019; Pichler and Lagnado, 2020). For example, by inhibiting the input of irrelevant information (e.g., constant flow of water across the body of a fish), the system becomes more sensitive to relevant stimuli (e.g., disruption of water flow caused by an approaching predator; von Holst and Mittelstaed, 1950).

**FIGURE 1:**
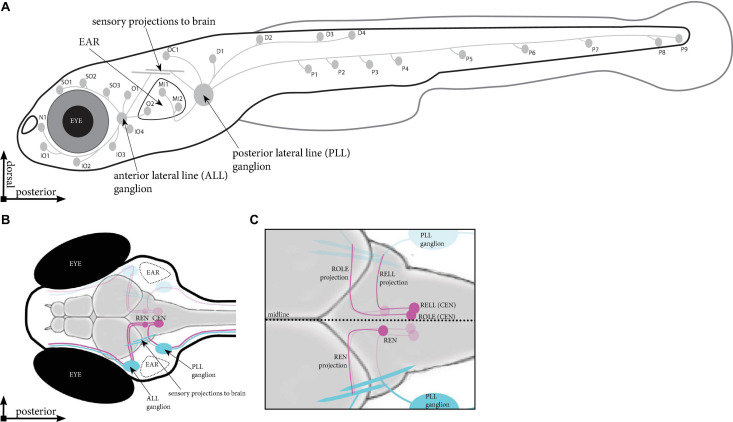
Schematic overview of the lateral line in zebrafish. **(A)** Location of the neuromasts in a zebrafish larva at 5 days post fertilisation (dpf). Indicated are the neuromasts identified in Tg(HGn39D), the anterior and posterior sensory ganglia and the projections they send to the neuromasts and into the brain. **(B)** Dorsal view of the head of a 5 dpf zebrafish larva. Indicated are the sensory ganglia and their projections (cyan) as well as the REN and CEN in the rhombencephalon (magenta), where we find the inhibitory efferent cells projecting to the lateral line. **(C)** Overview of the different cells in the REN and CEN (magenta). The REN contains a single cell type, which sends a projection anteriorly that turns toward the ALL ganglion. The CEN contains two cell types; ROLE, which projects past and follow the projection path of the REN cell; and a RELL cell, which projects till the REN cell, where it turns toward the PLL ganglion. ALL, anterior lateral line; PLL, posterior lateral line; REN, the rostral efferent nucleus; CEN, the caudal efferent nucleus nucleus; ROLE, the rhombencephalic octavolateral efferent neuron; RELL, the rhombencephalic efferent neuron to the lateral line.

To understand the formation and function of such a sensory circuit we study the lateral line, which is used by aquatic animals to detect water flow. The lateral line system consists of numerous sensory organs, neuromasts, which are typically arranged in superficial lines covering the head and body. The neuromasts are innervated by sensory neurons situated in two ganglia, giving rise to two separated networks: the anterior lateral line (ALL) projecting around the head and the posterior lateral line (PLL) projecting along the body (Gompel et al., 2001; Figure 1A). Neuromasts consist of hair cells that have cilia protruding from the skin, enabling the detection of water flow and play a crucial role in behaviors such as rheotaxis, predator avoidance, and schooling (Coombs et al., 2014; Olszewski et al., 2012). As water flows past the body, the protruding cilia bend, causing the release of glutamate (Pichler and Lagnado, 2019), which is detected by the sensory afferent projections. The information is then relayed to the brain (Vanwalleghem et al., 2020), so a proper behavioral response can follow.

The lateral line system is modulated by efferent neurons located in the brain. In zebrafish, modulatory efferent neurons can be found in six nuclei, three on each side of the midline: the diencephalic efferent of the lateral line (DELL) located in the diencephalon, and the rostral efferent nucleus (REN), and the caudal efferent nucleus (CEN) found in the rhombencephalon (Bricaud et al., 2001). DELL neurons are excitatory neurons that act upon the lateral line system via dopamine to increase the sensitivity of sensory input (Toro et al., 2015; Haehnel-Taguchi et al., 2018). In contrast, REN and CEN neurons are cholinergic and attenuate lateral line sensitivity (Flock and Russell, 1973), thereby reducing hair cell-induced activation of sensory afferent fibers. These flow-sensing hair cells are similar to those in the mammalian ear and several zebrafish models exist for the study of human hearing disorders (Whitfield, 2002). The lateral line system has also been used to identify multiple drugs and drug-like compounds that protect against hair cell death (Coffin et al., 2010; Ou et al., 2010). A deeper characterization of how efferent cells modulate sensory perception will increase our understanding of the hair cell circuitry and provide new opportunities to study the cause of related human diseases.

A recent study provided an anatomical overview of the projections made by neurons in the DELL nuclei (Haehnel-Taguchi et al., 2018). Their data revealed peripheral DELL projections to all larval anterior and posterior lateral line neuromasts. They also show that dopamine modulation of sensory information was not limited to the neuromasts, but it also directly targets the lateral line ganglia. Previous studies have revealed the location of the REN and CEN and, in part, described their projection paths (Zottoli and Van Horne, 1983; Higashijima et al., 2000; Bricaud et al., 2001; Figure 1B). One cell type was described for the REN and two cell types, with unique projection paths, were documented for the CEN: the rhombencephalic octavolateral efferent neuron (ROLE) and the rhombencephalic efferent neuron to the lateral line (RELL; Bricaud et al., 2001; Figure 1C). To complement these previous studies, we used transgenic zebrafish larvae, labeling inhibitory efferent neurons in a mosaic manner, to provide an anatomical overview of projections made by individual inhibitory efferent cells. Our data suggest that there are six inhibitory efferent cells on each side of the midline: two in the REN, and four in the CEN (two ROLE and two RELL cells). Cells in the REN primarily projected to the ALL whereas cells in the CEN projected to the ALL and PLL. Moreover, individual inhibitory efferent projections connected to 8–10 neuromasts and single neuromasts were innervated by multiple inhibitory efferent cells. Lastly, we observed that inhibitory efferent projections grow along the lateral line nerve at the same rate as sensory afferents (1 μm/min), but follow approximately 8–14 h after the primary sensory afferent projection. Our data bring new insights into the formation of the lateral line network, suggesting functional differences between the REN and CEN; serving as a stepping stone for functional studies aimed at unraveling how efferent signaling modulates sensory perception.

## Results and Discussion

### Mapping Inhibitory Efferent Projections to the Lateral Line

**FIGURE 2:**
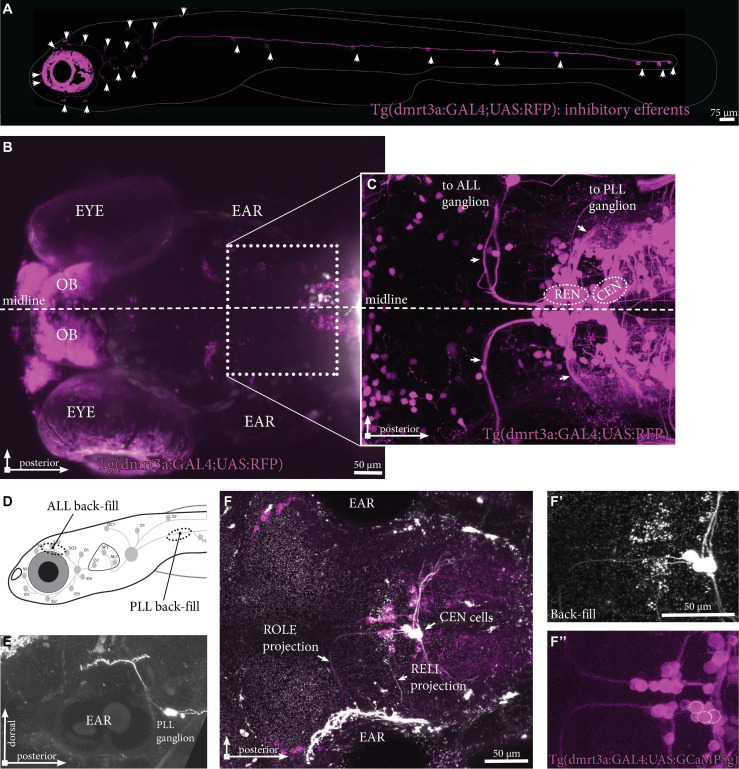
Inhibitory efferent projections to the lateral line in zebrafish larvae. **(A)** Overview of the inhibitory efferent projections in a Tg(*dmrt3a*:GAL4;UAS:RFP) 5 dpf larvae. Arrowheads indicate sites of neuromast innervation. Note that this is not the original confocal image, but a modified version to show the inhibitory efferent projections only; for the original image, please see Supplementary Figure 1. **(B)** Top view showing the head of the same larvae. Features marked are the eye, the ear and the olfactory bulb (OB; RFP-positive). Boxed area is shown in **(C)**. **(C)** Confocal image showing the REN and CEN in the hindbrain. Arrows indicate projections to the ALL ganglion and the PLL ganglion. **(D)** Schematic overview to indicate sites of ALL (S01-SO3; above the eye) and PLL (P1, above the yolk) back-fills. **(E)** Side view of a PLL back-fill. Back-fills were performed posterior of the PLL ganglion. Note the dendritic process from the ganglion to the brain (above the ear). **(F)** Overlay of back-filled inhibitory efferent cells in the CEN with the cells labeled by Tg(*dmrt3a*:GAL4;UAS:GCaMP5g). **(F’)** Back-fill of ROLE and RELL cells located in the CEN. **(F”)** Location of overlap by back-filled cells are indicated by white dotted circles in a Nacre Tg(*dmrt3a*:GAL4;UAS:GCaMP5g) larva. OB, olfactory bulb; ALL, anterior lateral line; PLL, posterior lateral line; REN, the rostral efferent nucleus; CEN, the caudal efferent nucleus nucleus; ROLE, the rhombencephalic octavolateral efferent neuron; RELL, the rhombencephalic efferent neuron to the lateral line.

The inhibitory efferent cells in this study were labeled by the *doublesex and mab-3 related transcription factor 3* (*dmrt3a)* promotor [Tg(*dmrt3a*:GAL4); Satou et al., 2020; Figure 2A]. In this transgene we were able to back-trace projections innervating the lateral line to cell bodies located in the REN and CEN (Figures 2B,C). To reveal if the transgenic line labels all inhibitory efferent cells, we performed back-fills using Texas Red labeled Dextran in Tg(*dmrt3a*:GAL4;UAS:GCaMP5g) larvae (Figures 2D,F). Back-fills were performed for the PLL (at the P1 neuromast) in transgenic larvae (Figure 2D). The REN and CEN were imaged in larvae that displayed back-filled cells in the sensory ganglia (Figure 2E). All inhibitory efferent cells labeled by back-fills were also labeled by the transgenic line (24 cells in 13 larvae; Figures 2F,F’,F”), indicating that the transgenic line labels all efferent cells innervating the lateral line.

#### Individual Inhibitory Efferent Projections

**FIGURE 3:**
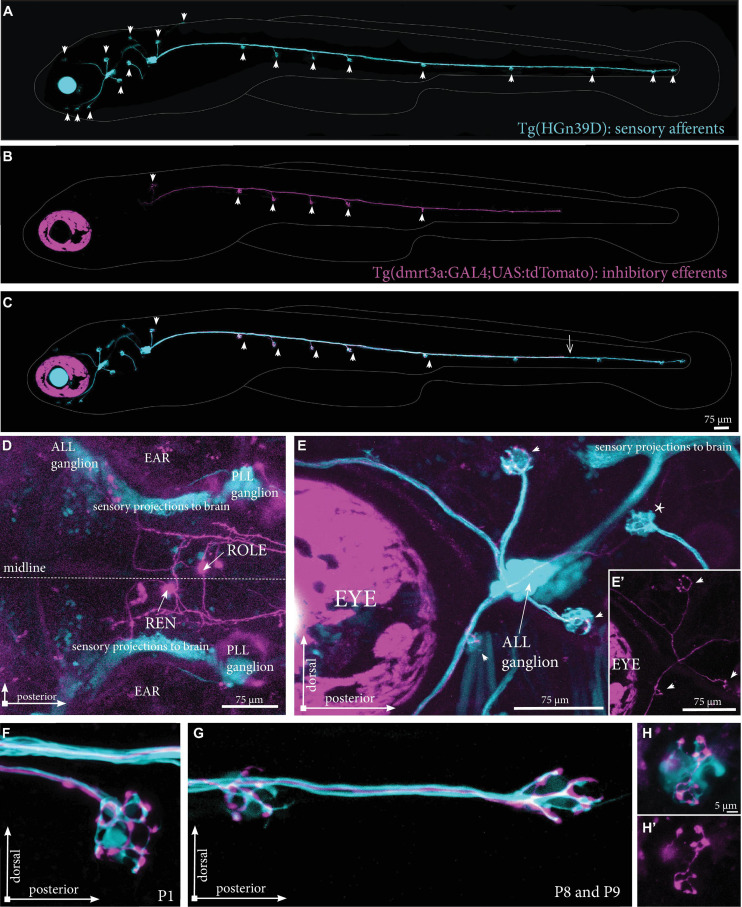
Projections by single inhibitory efferent cells. **(A)** Overview of the sensory afferent projections in Tg(HGn39D) at 5 dpf. Arrowheads indicate sites of neuromast innervation. **(B)** A single labeled inhibitory efferent neuron in Tg(*dmrt3a*:GAL4;UAS:tdTomato) showing partial coverage of the lateral line by its projections. Arrowheads indicate sites of neuromast innervation. **(C)** Overlay of A and B. Arrowheads indicate sites of afferent and efferent innervation of neuromasts. Arrow indicates the growth cone of the inhibitory efferent projection. Note that A-C do not show the original confocal image, but a modified version to show the lateral line projections only; for the original images, please see Supplementary Figure 2. **(D)** Top view of a Tg(*dmrt3a*:GAL4;UAS:tdTomato), where only a single cell in the REN and a single cell in the CEN (ROLE) can be seen. **(E)** Zoomed image of the ALL ganglion of Tg(HGn39D; *dmrt3a*:GAL4;UAS:tdTomato) reveal a single projection along the lateral line nerve of the ALL. Arrowheads indicate sites of neuromast innervation by the inhibitory efferent. Asterisks indicate neuromasts without inhibitory efferent innervation [**(E’)** shows the inhibitory efferent projection only]. **(F–G)** Higher magnification of Tg(HGn39D; *dmrt3a*:GAL4;UAS:tdTomato), showing the sensory afferents and a single efferent projection innervating a neuromast at P1 **(F)** and at P8 and P9 **(G)**. **(H)** Example of a neuromast that is only partially innervated by the single inhibitory efferent [**(H’)** shows the inhibitory efferent projection only]. ALL, anterior lateral line; PLL, posterior lateral line; REN, the rostral efferent nucleus; ROLE, the rhombencephalic octavolateral efferent neuron; RELL, the rhombencephalic efferent neuron to the lateral line.

To overcome difficulties in distinguishing the tightly clustered cell bodies and overlapping projection paths we used Tg(*dmrt3a*:GAL4;UAS:tdTomato) that express tdTomato in a mosaic manner to sparsely label cells of the REN and CEN. By crossing this line with Tg(HGn39D), which labels all sensory afferent cells with eGFP, we could assess the projections made by individual inhibitory efferent cells in relation to the sensory cells (Figures 3A–C). We screened the REN and CEN for larvae hosting one or two tdTomato-positive inhibitory efferent cells and traced their projections throughout the lateral line (Figures 3D–G). In the Tg(HGn39D) background, we were able to accurately map neuromasts and determine if they were innervated by inhibitory efferent projections. We could also reveal that the efferent cells project to the sensory ganglia where the process branch to follow the different tracts of sensory afferent projections toward the neuromasts (Figures 3E,E’). Imaging inhibitory efferent projections at the level of neuromasts revealed clear innervation of the hair cells and synaptic buttons throughout the lateral line (Figures 3F,G). Such detailed imaging revealed that the area innervated by an inhibitory efferent projection was in some cases less compared to that of the sensory afferent (Figures 3H,H’).

**FIGURE 4:**
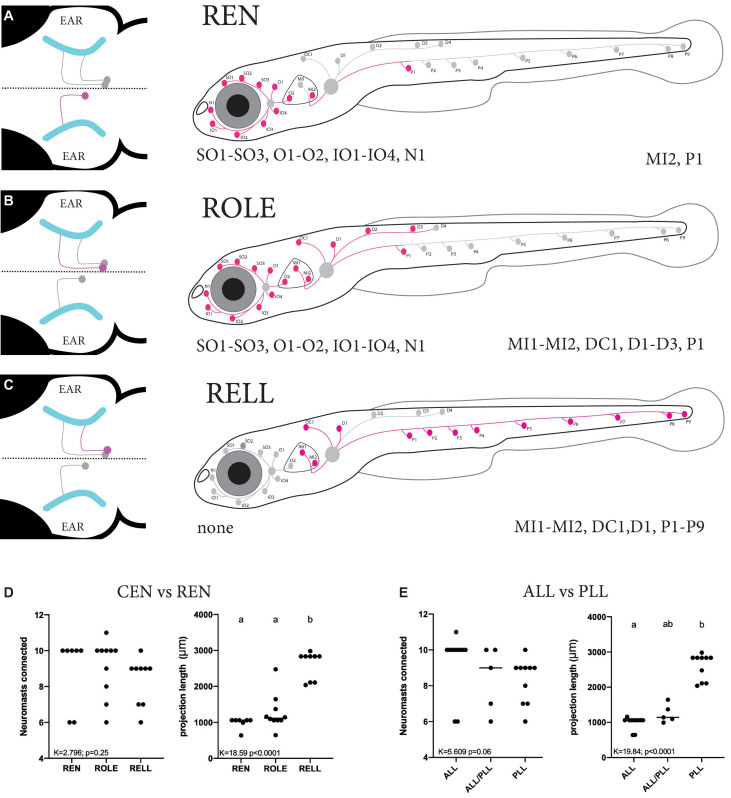
Summary of the projection paths of individual inhibitory efferent cells. **(A–C)** Schematic zebrafish larvae showing the cumulative projection paths of REN (*n* = 8), ROLE (*n* = 10), and RELL (*n* = 9) cells. Left drawing illustrates the projection paths of the REN, ROLE, and RELL cells (highlighted in magenta) within the rhombencephalon; projections of the sensory ganglion into the brain are shown in cyan. For each inhibitory efferent the cumulative projection path across the lateral line is highlighted (magenta) in the schematic zebrafish larvae on the right (for individual processes, please see Supplementary Figure 3). Identity of innervated neuromasts is listed below each schematic (left: anterior lateral line neuromasts; right: posterior lateral line neuromasts). **(D)** Quantification of the number of neuromasts connected revealed no significant differences between REN, ROLE, and RELL cells (*K* = 2.789, *p* = 0.25). The total length of the projections made by the RELL cells was significantly longer compared to that of the REN and ROLE cells (*K* = 18.59, *p* < 0.0001). **(E)** No significant differences were found between the number of neuromasts connected by individual inhibitory efferent cells that project to the ALL only, ALL and PLL, or PLL only (*K* = 5.609, *p* = 0.06). The total length of the projections made by cells that connect in the PLL was significantly longer compared to those connecting to the ALL but not the ALL+PLL (*K* = 16.84, *p* < 0.0001). Age of larvae: 5dpf. ALL, anterior lateral line; PLL, posterior lateral line; REN, the rostral efferent nucleus; CEN, the caudal efferent nucleus nucleus; ROLE, the rhombencephalic octavolateral efferent neuron; RELL, the rhombencephalic efferent neuron to the lateral line.

The occurrence of larvae with only a single cell were scarce, the majority of larvae either had too many or no tdTomato-positive inhibitory efferent cells, making data collection challenging. In addition, some types of inhibitory efferent cells (e.g., ROLE) were more commonly seen than others (e.g., RELL). We traced and mapped the projection patterns of 27 individual cells located in the REN and CEN (Supplementary Figure 3). We overlaid these individual projections to generate a “cumulative” map of the neuromasts found connected to either REN, ROLE, or RELL cells. We found only a single cell type located in the REN that always projected to the ALL (8/8) (Figure 4A and Table 1). In two cases we observed projections innervating the first part of the PLL in addition to the ALL (2/8) as the axon bifurcated at the level of the ear. For the CEN we observed two cell types: the ROLE cell (Figure 4B) and the RELL cell (Figure 4C), which were identified by their initial projection paths toward the sensory ganglia (Bricaud et al., 2001). The ROLE cells projected to either the ALL (5/10), the PLL (1/10), or to both (4/10) with the axon bifurcating at the level of the ear. In contrast, the RELL cells were found to only project to the PLL (9/9) (Figure 4C and Table 1). While excitatory efferent projections have been found to innervate the sensory ganglia (Haehnel-Taguchi et al., 2018), this was not observed among the inhibitory efferent projections described here. However, it should be noted that we selected for larvae with inhibitory efferent projections to neuromasts and cannot exclude that other classes of inhibitory efferent cells, located in the REN or CEN, project to the sensory ganglia or other organs in the periphery.

**TABLE 1:** Number of REN, ROLE, and RELL cells that project to the anterior lateral line (ALL), posterior lateral line (PLL), or both.

	**ALL**	**ALL/PLL**	**PLL**
REN (*n* = 8)	6 (75%)	2 (25%)	0 (0%)
ROLE (*n* = 10)	5 (50%)	4 (40%)	1 (10%)
RELL (*n* = 9)	0 (0%)	0 (0%)	9 (100%)

*Number of inhibitory efferent cells projecting to the ALL and PLL.*

To get a better understanding of the number of inhibitory efferent cells projecting to the ALL and PLL, we grouped our cells based on their innervation of the ALL or PLL. We found that 17 cells projected to the ALL: 8 located in the REN (47%) and 9 located in the CEN (53%). For the PLL we had 16 projecting cells: 2 located in the REN (12%) and 14 located in the CEN (88%) (Table 2: first column).

**TABLE 2:** Distribution in the REN and CEN of analyzed inhibitory efferent cells innervating the anterior lateral line (ALL) or posterior lateral line (PLL).

		**Transgenic**	**Back-fill**	Back-fill*	**Average**
ALL innervation	REN	47%	60%	42%	50%
	CEN	53%	40%	58%	50%
PLL innervation	REN	12%	22%	8%	14%
	CEN	88%	78%	92%	86%

*Bricaud et al. (2001).

Next, we performed back-fill experiments to support our findings in the transgenic line (Figure 2D). For cuts in the ALL we observed labeling in the REN in 11 out of 13 (1–2 cells per larvae) and we found cells in the CEN in 6 out of 13 cases (1–2 cells per larvae). In total we counted 20 cells, of which 12 were positioned in the REN (60%). In one larva we observed 2 REN cells. For cuts made to the PLL we obtained 11 back-fills where we were able to assign cells to the REN (6/11; 1 cell per larvae) and CEN (11/11; 1–4 cells per larvae). Here we identified a total of 27 cells, of which 6 where found in the REN (22%) and 21 in the CEN (78%) (Table 2: second column). In four larvae we identified 2 ROLE cells and in one larva we found 2 RELL cells. Although the existence of two RELL cells has been reported (Metcalfe et al., 1985), the observation of 2 REN cells and 2 ROLE cells has not previously been described. Data from the transgenic line, our back-fills, and back-fills from a previous study (Bricaud et al., 2001; Table 2: third column), were all in line with each other.

We noticed that fewer cells projected to the P8-P9 neuromasts of the PLL compared to the number of cells connecting to the P1 neuromast in the transgenic line. To confirm this, we performed back-fills near the last two neuromasts of the PLL. We obtained 10 successful back-fills in which we identified a total of 11 cells (1–2 cells per larvae) that had projections to the end of the PLL. We found that all cells were located in the CEN (100%), compared to 78% when the cut was made at P1. In back-fills made close to the P1 neuromast, 11/21 cells were classified as RELL (52%), compared to 11/11 (100%) in the P8-P9 back-fill. These observations support our finding using the transgenic line, were no REN or ROLE cells connected to the P8-P9 neuromasts (see Figures 4A–C).

The data obtained from the transgenic line are in accordance with previous conclusions drawn from back-fills: cells in REN favor projecting to the ALL, while cells in CEN project to the ALL and PLL in equal proportion (Bricaud et al., 2001). However, our transgenic data shows that a larger number of inhibitory efferent cells connect to both the ALL and PLL than previously described. For example, we found that 6 (2 REN and 4 CEN) out of the 27 cells had projections to both the ALL and PLL. This is a relatively high percentage compared to previous studies reporting a lack of double labeled cells in the REN or CEN following simultaneous back-fills of both the ALL and PLL (1/76 cells; Wagner and Schwartz, 1996 and 0/77 cells; Bricaud et al., 2001). Methodological differences may underlie the contrasting results, where the use of a transgenic line is advantageous compared to back-fills. During back-fills the lateral line projections are generally exposed to dye at the level of SO1-SO3 (for ALL) and P1 (for PLL). If we look at the single cell projection paths in all our transgenic fish, we find that out of the 27 cells analyzed, only a single cell had projections that crossed both these sites. This means, had we used back-fills, we would have missed 5 out of the 6 inhibitory efferent neurons that project to both the ALL and PLL.

#### Innervation of Neuromasts by the Inhibitory Efferent Projections

As the projection paths of individual REN, ROLE, and RELL cells revealed some variation in their innervation, we quantified the number of neuromasts they connect to and the length of their projection paths. There was no significant difference in the number of neuromasts innervated by REN (6–10 neuromasts), ROLE (6–11 neuromasts), and RELL (6–10 neuromasts) cells (*K* = 2.796; *p* = 0.25; Figure 4D). Next, we estimated the lengths of individual inhibitory efferent projections by quantifying the length traveled along the lateral line nerve. A Kruskal Wallis test revealed that the length of the projection path made by RELL cells (2,602 ± 391 μm) was significantly longer compared to the paths made by the ROLE cells (1,271 ± 494 μm) and REN cells (991 ± 157 μm) (*K* = 18.50 *p* < 0.0001; Figure 4D).

Next, we analyzed the same parameters with groups based on projections to the ALL and/or PLL. Here too, we found no significant differences in the number of innervated neuromasts (6–11 neuromasts) by cells projecting to the ALL and/or the PLL (*K* = 5.609; *p* = 0.06). The length of projections to the PLL (2,590 ± 371 μm) were longer compared to those toward the ALL/PLL (1,249 ± 261 μm; *p* = 0.055) and significantly longer compared to ALL only projections (993 ± 176 μm; *p* < 0.0001) (Kruskal Wallis test, *K* = 17.54; *p* = 0.0002; Figure 4E). In all, our data show that projections that only covered the PLL had a significantly longer path than those covering the ALL or ALL/PLL but that they innervate the same number of neuromasts. The difference in projection lengths is not surprising as the PLL covers the body of the zebrafish larvae, which is several times larger than the head that is covered by the ALL.

### The Growth of Inhibitory Efferent Processes Along the Posterior Lateral Line

The growth cone of the primary sensory afferent closely follows the neuromast primordium migrating along the body during development (Metcalfe, 1985; Ghysen and Dambly-Chaudière, 2004). To explore if this is also the case for efferent projections we conducted a series of time-lapse recordings of Tg(HGn39D; *dmrta*:GAL4;UAS:RFP) embryos.

#### Time-Lapse Imaging of Posterior Lateral Line Innervation

**FIGURE 5:**
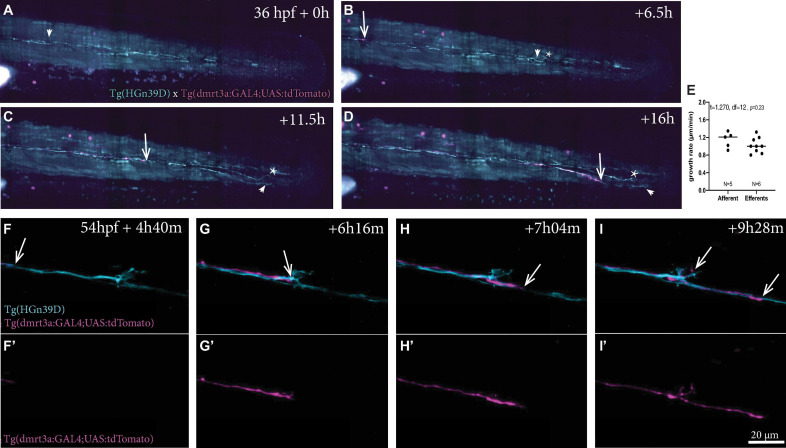
Time-lapse recordings of sensory afferent and inhibitory efferent processes growing along the PLL. **(A–D)** Time frames of time-lapse imaging made by digital light-sheet microscopy in a Tg(HGn39D; *dmrt3a*:GAL4;UAS:tdTomato) larva. Arrowheads indicate the growth cone for the sensory afferent projection and arrows indicate the growth cone of the inhibitory efferent projection. Asterisks indicates projections on the other side of the larvae’s body visible in the maximum intensity projections. **(E)** The growth rate between the sensory afferent projections (1.15 ± 0.18 μm/min) and inhibitory efferent projections (1.0 ± 0.17 μm/min) was not significantly different (*t* = 1.581, *df* = 14, *p* = 0.14). **(F–I)** Higher magnification time-lapse confocal imaging of the synaptic terminals of afferent processes and a single inhibitory efferent process [**(F’–I’)** show inhibitory efferent projection only]. Recordings show innervation of a neuromasts by sensory afferents prior to inhibitory efferent innervation. Arrows indicate the growth cone of the inhibitory efferent. Age of larva in **(A)** 36 hpf; Age of larva in **(F)** is 54 hpf.

Using light sheet microscopy, we generated 16-h time-lapse recordings during the growth of sensory afferent and inhibitory efferent projections in embryos starting at 36 h post fertilisation (hpf) (Figures 5A–D and Supplementary Video 1). We found that the inhibitory efferent projections did not travel with the primordium as the primary sensory afferent projection, but followed later at a distance of 690 ± 80 μm (*n* = 3), which corresponds to an 8–14 h delay. It has been shown that for some developing circuits, primary neurons, termed pioneers, can lay down an axonal scaffold that allows follower axons to grow at a faster rate (Bak, 2003). We therefore compared the growth rates of sensory afferent projections (1.15 ± 0.18 μm/min; *n* = 5) to the inhibitory efferent projections (1.0 ± 0.17 μm/min; *n* = 9), but found no significant differences (*t* = 1.270, *df* = 12, *p* = 0.23; Figure 5E). In addition, in 2 out of 6 recordings we observed a second inhibitory efferent projection growing across the lateral line nerve at a later time-point. When we compared growth rates between the first and second inhibitory efferent projection, we found no differences (*t* = 0.4082, *df* = 6, *p* = 0.70). These observations are in line with previous findings regarding sensory lateral line projections, where there was no difference in growth rate between the primary and following projections (1.33 ± 0.13 μm/min; Sato et al., 2010). Combined, our results suggest that, if there is such a thing as pioneer neurons for the lateral line, then they do not increase the growth rate of follower neurons. Whether the absence of a primary afferent projection affects the growth rate of follower projections, as for example seen with motor neurons in zebrafish (Pike et al., 1992), remains to be investigated.

Higher magnification time-lapse imaging allowed us to visualize the innervation of a single neuromast by inhibitory efferent projections (Supplementary Videos 2–5). In all recordings, we found that inhibitory efferent projections closely followed the path of the sensory afferents and bifurcation occurred at the site where sensory afferents split off from the lateral nerve to project toward a neuromast. In some recordings, branching occurred several hours after the inhibitory efferent growth cone had passed the neuromast (Figures 5F,F’–I,I’; see Supplementary Videos 2, 3). As axon guidance plays an important role in bifurcation (Lewis et al., 2013), the delay might be caused by the restructuring required to respond and react to the attractive cues originating from the neuromasts. This delay was not observed for innervation of the P8 and 9 neuromasts, possibly a result of them representing the end of the lateral line (Supplementary Video 4). Occasionally we observed sensory afferents sending projections out from a neuromast, which seemed to be growing in parallel to (or even toward) the lateral line nerve (Supplementary Video 5).

### Multiple Inhibitory Efferent Cells Innervate a Single Neuromast

While we have shown that individual inhibitory efferent projections connect to multiple neuromasts (Figures 3, 4), we were also interested in if multiple inhibitory efferent cells connect to the same neuromast. We found at times that the innervation of neuromasts by individual projections covered a smaller area than that of the sensory afferent innervation (Figure 3H). This could either be due to delayed development (i.e., hair cells are yet to be connected by the projection) or to the existence of a second (unlabelled) inhibitory efferent projection covering that space. Our mapping data (Figure 4) supports the idea of two (or more) inhibitory efferent cells innervating a single neuromast as both REN and ROLE cells were mapped to the same neuromasts in the ALL, and similarly for ROLE and RELL cells and PLL neuromasts.

**FIGURE 6:**
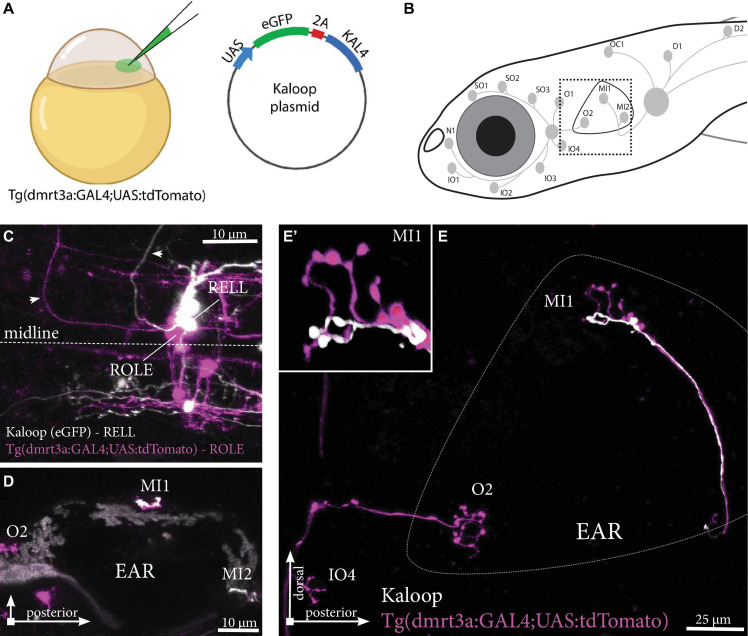
Tg(*dmrt3a*:GAL4;UAS:tdTomato) injected with Kaloop plasmid reveal multiple inhibitory efferent cells innervating a single neuromast. **(A)** One-cell stage embryos of Tg(*dmrt3a*:GAL4;UAS:tdTomato) were injected with Kaloop plasmid to generate larvae with sparsely labeled tdTomato and eGFP inhibitory efferents. **(B)** Schematic overview of the area shown in **(E)**. **(C–D)** Confocal images showing the view of sparsely labeled inhibitory efferent cells in their nuclei. A ROLE (tdTomato-positive; magenta) and a RELL (eGFP-positive; white) cell were identified **(C)** and their projections traced across the lateral line **(D)** dorsal view and **(E)** lateral view of the inhibitory efferent projections at the level of the ear. In this case, both efferent cells were connected to the same neuromast located on the ear (MI1) [**(E’)** depicts the MI1 neuromast at higher magnification]. Age of larvae: 5 dpf. ROLE, the rhombencephalic octavolateral efferent neuron; RELL, the rhombencephalic efferent neuron to the lateral line. **(A)** was partially created with BioRender.com

A low concentration of Kaloop plasmid, carrying both eGFP and KAL4 under the UAS-promoter, was injected in the cell of one-cell stage embryos of Tg(*dmrt3a*:GAL4;UAS:tdTomato) (Figure 6A). This generated larvae with sparsely labeled inhibitory efferent cells expressing eGFP and/or tdTomato. We identified larvae with neuromasts innervated by two, non-overlapping, inhibitory efferent projections. For instance, we observed a ROLE and a RELL cell both connected to the same MI1 neuromast of the PLL (Figures 6B–E). The ROLE cell connected to more neuromasts of the ALL, while the RELL cell innervated more neuromasts of the PLL (full projection paths not shown).

**FIGURE 7:**
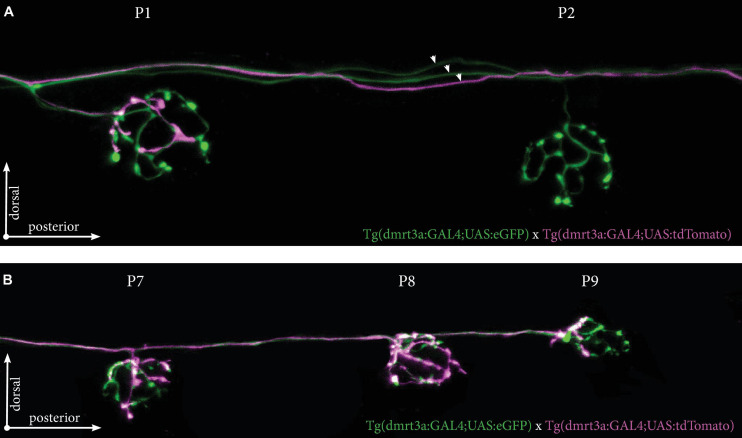
Tg(*dmrt3a*:GAL4;UAS:tdTomato) crossed with Tg(*dmrt3a*:GAL4;UAS:eGFP) reveal multiple inhibitory efferent processes innervating a single neuromast. **(A)** Confocal images of neuromast P1 and P2 of the PLL. All efferent projections were labeled by Tg(dmrt3a:GAL4;UAS:eGFP) (green) and single efferent projection was labeled by Tg(dmrt3a:GAL4;UAS:tdTomato) (magenta). Note how the single tdTomato-positive (magenta) projection connects to the P1 neuromast, but skips the P2 neuromast. Arrowheads indicate three different efferent projections along the PLL. **(B)** In the same larvae as in **(A)**, the single tdTomato-positive (magenta) projection connects to the last three neuromasts of the PLL (P7-P9). Age of larvae: 5 dpf.

We also crossed Tg(*dmrt3a*:GAL4;UAS:eGFP), which labels all inhibitory efferent cells to Tg(*dmrt3a*:GAL4;UAS:tdTomato), which generates mosaic expression. As one example, we identified a larva with at least three different inhibitory efferent projections (two eGFP-positive and one eGFP/tdTomato-positive) projecting along the PLL (Figure 7A). We found that the P1 neuromast was connected by both eGFP- and tdTomato-postive projections, revealing it to be innervated by at least two inhibitory efferent cells. The tdTomato-positive projection only covered a portion of the neuromast (approximately 25% of the area) suggesting partial innervation. Interestingly, although the P2 neuromast was not innervated by the tdTomato-positive projection, it did continue to project along the lateral line and innervated the downstream P7, P8, and P9 neuromasts (Figure 7B).

#### Functional Implication of Innervation by Multiple Efferent Cells

The observation that neuromasts are innervated by multiple inhibitory efferent cells supports the hypothesis that there are differences in function between the cells found in the REN and CEN (Bricaud et al., 2001). For example, the projections of RELL cells come in close contact with the projection of the Mauthner cell (Metcalfe et al., 1985), which is the main cell driving fast-escape responses (Faber et al., 1989; Tabor et al., 2014). Others have shown that activation of neuromasts induce an escape response (McHenry et al., 2009; Pichler and Lagnado, 2019) and that there is a monosynaptic connection between the sensory afferents and the Mauthner cell (Mirjany and Faber, 2011). Based on these observations it is possible that RELL cells are involved in feedforward inhibition of neuromast during escape behaviors. A drastic change in water flow will register a strong response in the sensory afferent, sufficient to activate an escape response triggered by the Mauthner cell, which in turn, via activation of the RELL cell, attenuates lateral line sensitivity to prevent overloading the sensory network with self-inflicted stimuli. Thus, REN and ROLE cells could be more crucial for other types of feedforward inhibition, e.g., normal swim episodes (Lunsford et al., 2019; Pichler and Lagnado, 2020), or events where feedback inhibition is required, e.g., exposure to a constant water flow. Further studies are required to explore these hypotheses.

## Concluding Remarks

The zebrafish lateral line represents a small sensory circuit where the individual components can be analyzed in detail to understand how sensory information is processed and modulated. Three different classes of inhibitory efferent cells innervate the sensory hair cells to provide feedforward and feedback inhibition. Through transgenic labeling, we provide an anatomical overview of the projections made by single inhibitory efferent cells connected to the lateral line, revealing unique projection patterns. A previous study reported the existence of two RELL cells in the CEN (Metcalfe et al., 1985) something we also observed. In addition, we identified two ROLE and two REN cells in individual larvae. REN cells project to the anterior lateral line, RELL cells project to the posterior lateral line and ROLE cells project to both. We show that a single inhibitory efferent cell connects to 6–10 neuromasts and that a single neuromast can be innervated by at least two inhibitory efferent cells.

The dual REN, ROLE and RELL cells may provide orientation selective inhibition, similar to the split innervation by sensory afferents ensuring direction sensitivity (Faucherre et al., 2009). Alternatively, both may eventually connect to all hair cells in a neuromast, and thereby provide a stronger inhibitory signal. It is also possible that the two cells sharing a projection pattern have different inputs, thereby regulating hair cell sensitivity during different behaviors. Functional analyses and morphological characterization in older larvae or adult zebrafish should offer further insights into the formation and function of the inhibitory efferent cells innervating the lateral line. Our study represents a stepping-stone toward future studies where the sub-functionality of these different classes of inhibitory efferent cells can be addressed, so that their involvement in feedback and feedforward events coupled to various behaviors can be assessed. Anatomical and functional studies of this powerful model system will provide new opportunities to study the biology of sensory modulation and relate it to disease.

## Materials and Methods

### Experimental Animals and Transgenic Lines

Adult zebrafish were housed at the Genome Engineering Zebrafish National Facility (SciLife Lab, Uppsala, Sweden) under standard conditions of 14/10 h light/dark cycles at 28∘C. Appropriate ethical approvals were obtained from a local ethical board in Uppsala (C164/14 and 14088/2019).

The following transgenic lines were used: Tg(*dmrt3a*:GAL4) (Satou et al., 2020), Tg(HGn39D) (Faucherre et al., 2009), Tg(UAS:GCaMP5g), Tg(UAS:RFP), Tg (UAS:eGFP), and Tg(UAS:tdTomato). The expression of tdTomato was mosaic, likely due to random silencing of its UAS repeats (Akitake et al., 2011). Embryos and larvae were kept under constant darkness at 28∘C. To prevent pigmentation, 1-Phenyl-2-thiourea (PTU, 0.003% final concentration) was added at 24 h post fertilisation (hpf). In addition, larvae of the Nacre strain (Lister et al., 1999) were used for the back-fill experiments.

### Neuronal Tracing Through Back-Fills

Black anodized minutien pins (tip diameter 17,5 μm, AgnThos) and small pins cut from 25 μm diameter tungsten wire (Advent Research Materials) were loaded by dipping in a viscous solution of Texas Red labeled Dextran 3000MW (Fisher Scientific/Invitrogen, Waltham, Massachusettes, United States) (Fritzsch, 1993). At 5 days post fertilisation (dpf), Nacre larvae were anesthetised with tricaine (0.12 mg/ml) and transferred to a 2% agarose plate where excess water was removed with a fibreless paper tissue. Light scratching of the skin with a dye-loaded pin was sufficient to rupture the neural projection and allow the dye to back-fill to the cell bodies. After a recovery period of at least 5 h larvae were screened for red fluorescence in the lateral line sensory ganglia, indicating a successful back-fill. Confocal imaging of back-filled inhibitory efferent cells was performed during the 3 days following a back-fill (6–8 dpf). Back-fills were performed at three locations in the lateral line system: (1) at the P8-9 neuromasts to label efferent cells projecting to the terminal end of the PLL, (2) just anterior to the P1 neuromast to label all efferent cells projecting to the PLL and (3) between the SO1-SO3 neuromasts to label efferent cells projecting to the ALL.

### Double Mosaic Labeling of Efferent Neurons

For double mosaic labeling of inhibitory efferent cells, we injected 50 pg of Kaloop plasmid (UAS:eGFP-2A-KAL4) into the cell of one-cell stage Tg(*dmrt3a*:GAL4;UAS:tdTomato) embryos. GAL4 activates eGFP and KAL4, a GAL4 variant (Distel et al., 2009), in the Kaloop plasmid, generating a self-sustaining loop that labels cells with eGFP. Dilution and random inheritance during development generates mosaic eGFP labeling of inhibitory efferent cells on top of the already mosaic tdTomato labeled inhibitory efferent cells.

### Microscopy

All imaging was performed using a Leica SP8 confocal microscope (Leica Microsystems, Wetzlar, Germany). For light-sheet imaging a Leica DLS module was mounted on the Leica SP8. Larvae were mounted in low melting agarose (1.2% for confocal microscopy; 0.8% for light-sheet microscopy) and kept anesthetised by Tricaine (0.12 mg/ml) during image acquisition. Image acquisition and processing was done using Leica’s LasX software. Time-lapse recordings were processed and analyzed using Fiji. Static confocal images were taken using a 25x water objective, while confocal time-lapse was performed using a 63x glycerol objective; images were taken every 5–8 min. For light-sheet time-lapse recordings, images were taken every 8 min with a 10x objective.

### Statistical Analysis

Statistical analyses were performed using Prism 9 for MacOS (GraphPad Software, La Jolla, United States). Gaussian distribution of the data was determined by a Kolmogorov-Smirnov test. Differences among individual efferent neurons were assessed using the non-parametric Kruskal-Wallis test, followed by a Dunn’s multiple comparison test. A parametric unpaired Student’s *t*-tests (two-tailed) was used to compare the migration rates of the afferent and efferent projections. Number of replicates for each experiment are indicated in each figure. Statistical significance was set at *p* ≤ 0.05.

## Data Availability Statement

The raw data supporting the conclusions of this article will be made available by the authors, without undue reservation.

## Ethics Statement

The animal study was reviewed and approved by Swedish Board of Agriculture.

## Author Contributions

RM: experimental design, data acquisition, data analysis, data interpretation, and drafting the manuscript. AI: data acquisition and revising the manuscript. JH and HK: data acquisition, data analysis, revising the manuscript. HB: experimental design, data acquisition, data analysis, data interpretation, and revising the manuscript. All authors contributed to the article and approved the submitted version.

## Conflict of Interest

The authors declare that the research was conducted in the absence of any commercial or financial relationships that could be construed as a potential conflict of interest.
